# When Ingestion Is Actually Aspiration: Delayed Diagnosis of a Subglottic Foreign Body

**DOI:** 10.7759/cureus.111675

**Published:** 2026-06-28

**Authors:** Abigail L Contello, Abraham A Kassem, Nicole Favre, Michele M Carr

**Affiliations:** 1 Otolaryngology - Head and Neck Surgery, Jacobs School of Medicine and Biomedical Sciences, University at Buffalo, Buffalo, USA

**Keywords:** adolescent airway presentation, delayed foreign body diagnosis, laryngoscopic foreign body removal, pediatric otolaryngology emergency, subglottic obstruction

## Abstract

Airway foreign body (AFB) is commonly encountered in young children but may present atypically in adolescents, contributing to a delayed diagnosis. We report a case of a previously healthy 14-year-old male who presented after choking on a plastic water bottle cap with persistent throat discomfort but no respiratory distress. Initial evaluation, including plain radiographs, esophagram, and computed tomography (CT) of the chest, was unrevealing, and the patient was discharged after tolerating oral intake. He returned two days later with persistent symptoms and new-onset hoarseness. Re-evaluation of CT imaging with dedicated review of airway structures revealed a radiolucent foreign body within the subglottic airway. The patient underwent successful removal via direct laryngoscopy without complications. We highlight key diagnostic challenges in AFB, particularly radiolucent objects and subacute presentations in older children. Negative initial imaging and absence of overt respiratory distress may provide false reassurance. A high index of suspicion, careful image review, and early airway-directed evaluation are essential to avoid delayed diagnosis and associated complications.

## Introduction

Airway foreign body (AFB) aspiration is most commonly seen in children, with nearly 20% of cases occurring in those younger than three years [1]. Complications of undiagnosed AFB include atelectasis, pneumonia, bronchiectasis, hypoxia, and death [2]. Because patients may present asymptomatically or with nonspecific symptoms such as cough, dyspnea, or throat discomfort, diagnosis can be delayed, with over 40% of cases identified more than 24 h after aspiration [1,3]. In the absence of acute airway compromise, the diagnosis often relies on a high index of suspicion, a patient-reported history of aspiration, and careful radiographic and endoscopic evaluation.

## Case presentation

A 14-year-old previously healthy male presented to the emergency department (ED) after an apparent accidental ingestion of a plastic water bottle cap. The patient reported chewing on the cap, which he inadvertently inhaled and swallowed. Immediately following the event, he experienced a persistent foreign body sensation in his throat but denied choking, drooling, gagging, vomiting, chest pain, dyspnea, or dysphagia. He had not consumed food or liquids since the incident.

**Figure 1:**
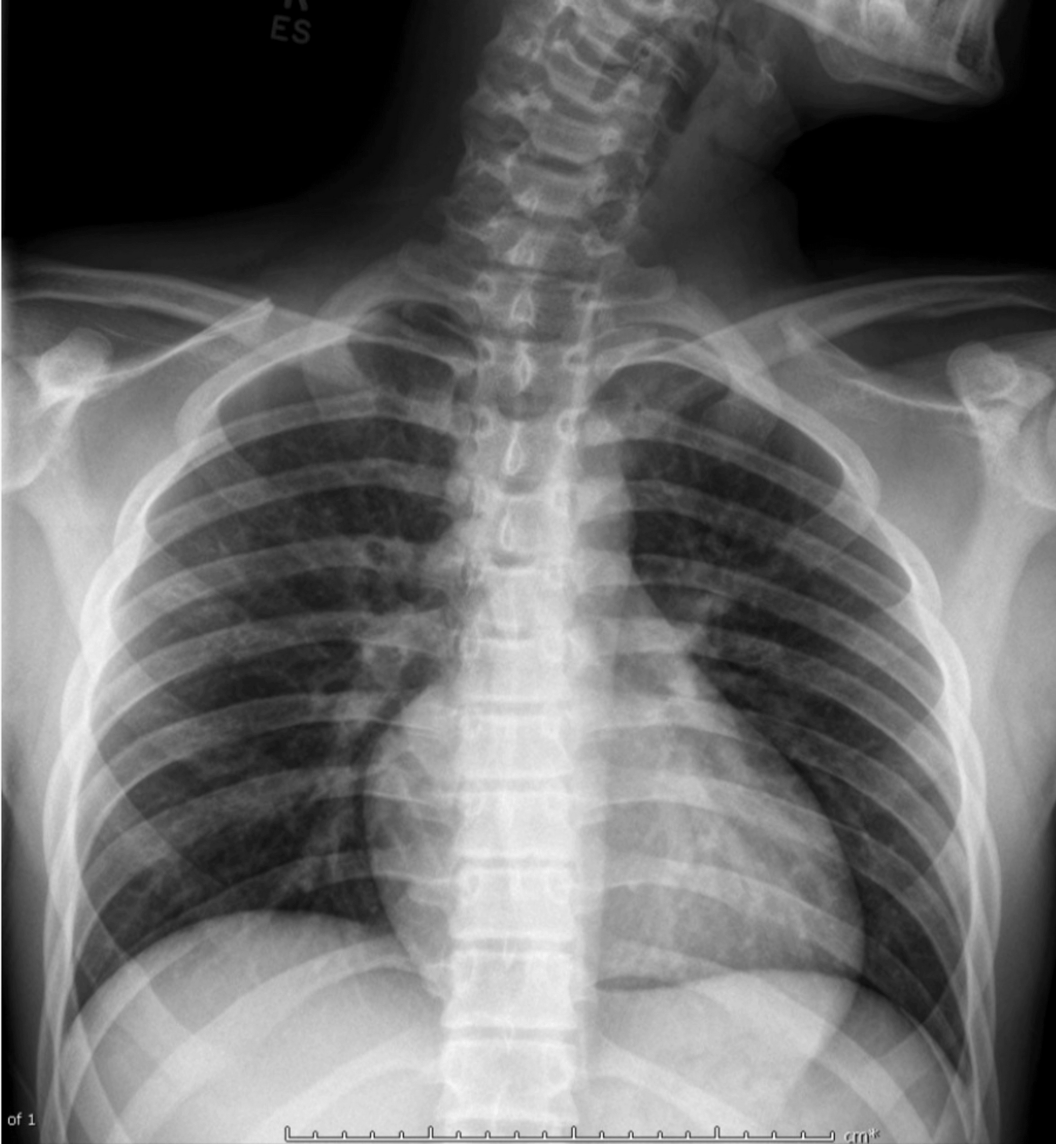
Initial plain, anteroposterior chest radiograph with negative findings.

On initial presentation, the patient was afebrile and hemodynamically stable. Physical examination by the ED team revealed a normal-appearing oropharynx without visible foreign body, mucosal injury, or pooling of secretions. Pulmonary examination demonstrated a bilaterally clear chest without evidence of increased respiratory effort, and the remainder of the examination was unremarkable. Plain anteroposterior radiographs from the mouth to rectum did not demonstrate a radiopaque foreign body or mediastinal widening (Figure 1). Given the absence of alarming clinical features and the likelihood that the object was ingested and was radiolucent, a supervised oral intake challenge was performed, which the patient tolerated without difficulty. He was discharged home by the ED staff with return precautions.

Two days later, the patient returned to the ED with persistent throat discomfort and a sensation of something “stuck” deeper in his throat. He also reported new-onset hoarseness and one brief episode of shortness of breath, which had since resolved. He denied ongoing dyspnea, chest pain, nausea, vomiting, abdominal pain, or difficulty swallowing. He was able to tolerate oral intake and reported normal bowel movements without passage of the foreign body.

Vital signs remained within normal limits, and the patient appeared comfortable without acute distress. Examination again demonstrated a normal oropharynx, supple neck without tenderness or lymphadenopathy, and clear lung fields with normal respiratory effort. Given persistent symptoms despite negative plain radiographs, further evaluation for a suspected esophageal foreign body was pursued.

A fluoroscopic esophagram was obtained and demonstrated normal esophageal caliber and motility without evidence of filling defect, obstruction, or retained foreign body. A noncontrast computed tomography (CT) scan of the chest was subsequently performed and was initially interpreted as unremarkable, without evidence of an esophageal foreign body.

**Figure 2:**
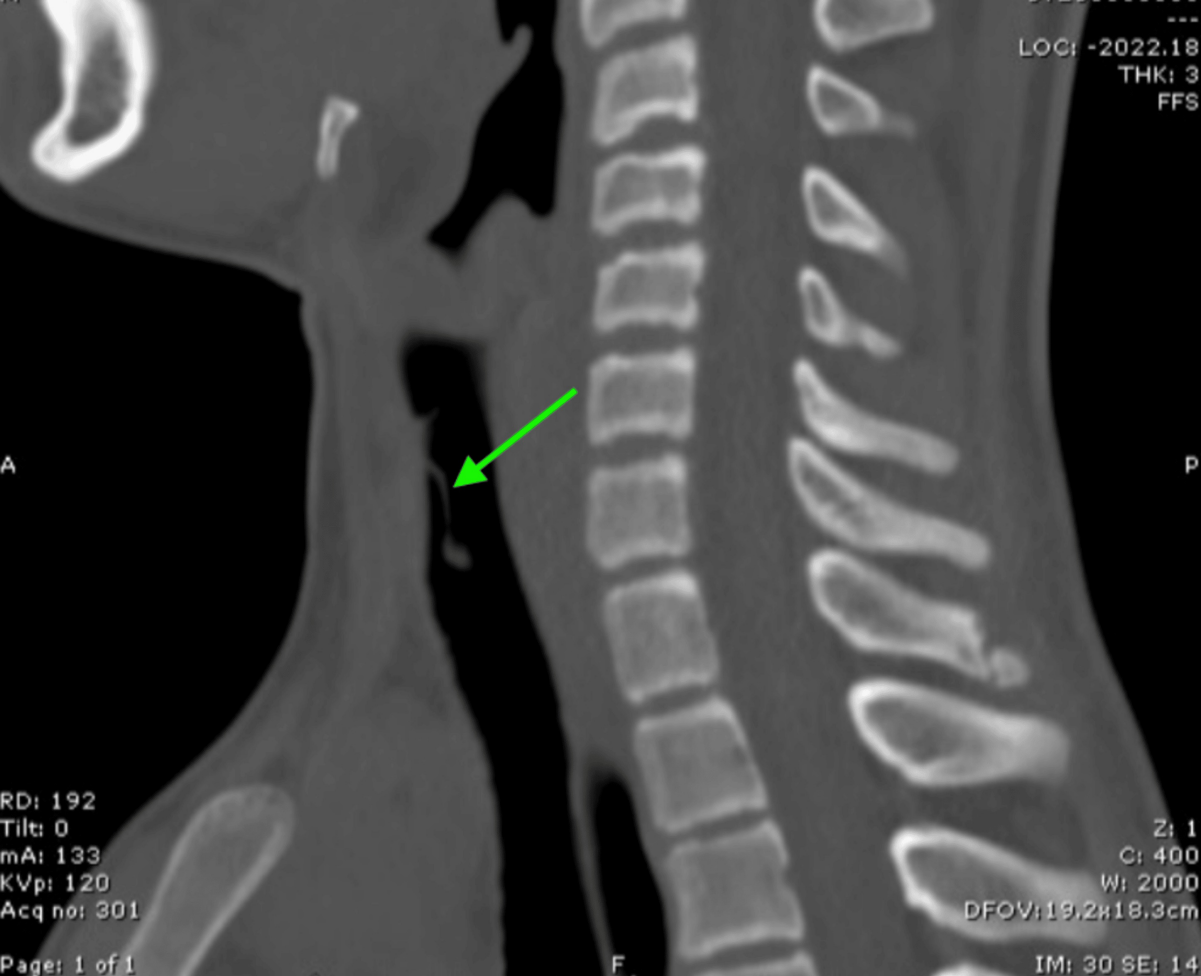
Noncontrast bone-window CT scan with arrow pointing to the foreign body in the subglottic airway.

The next morning, a subsequent review of the noncontrast CT scan of the neck revealed radiopaque material within the subglottic airway seen on bone windows, compatible with an aspirated plastic bottle cap (Figure 2). The trachea appeared to be of normal caliber, and no additional soft-tissue abnormalities of the neck were identified.

The otolaryngology team was consulted for airway foreign body management. The patient remained stable at the bedside, breathing comfortably on room air and tolerating secretions. Given the risk of airway compromise, the patient was admitted, kept NPO, and prepared for operative intervention. The possibility of tracheotomy in the setting of airway compromise was discussed with the patient and family.

**Figure 3:**
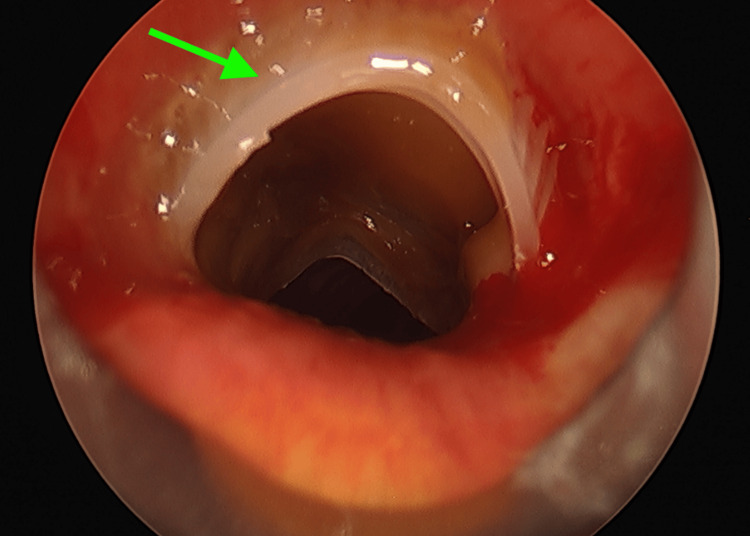
Bronchoscopic view with arrow pointing to plastic bottle cap in the subglottis. The bottle cap is folded against the anterior wall of the subglottis.

**Figure 4:**
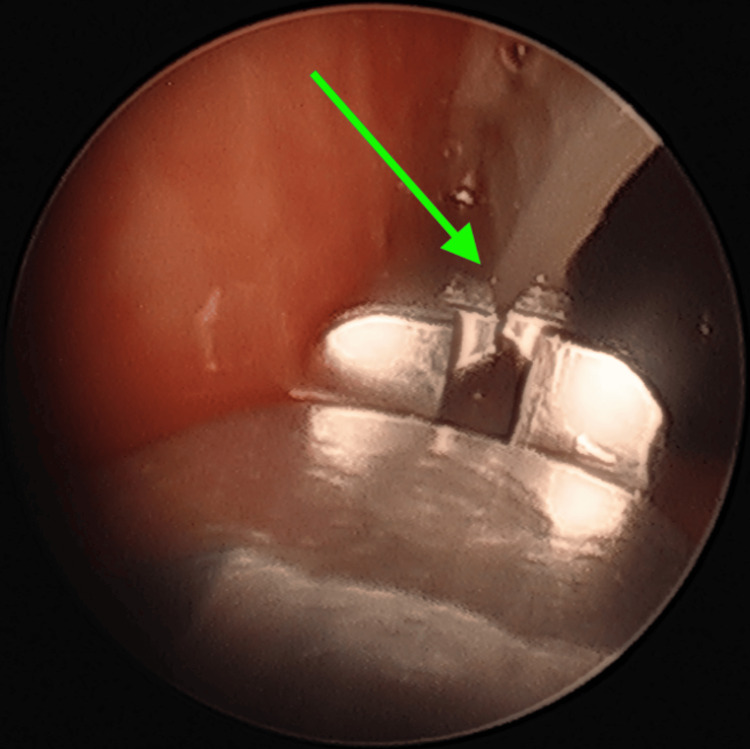
Bronchoscopic image with arrow pointing to extraction of AFB with optical alligator forceps. AFB: airway foreign body

The patient was taken to the operating room for direct laryngoscopy and bronchoscopy under general anesthesia. A large Parsons laryngoscope was used to access the larynx, and bronchoscopy demonstrated a clear plastic water bottle cap measuring 28 mm in diameter and 6 mm in height lodged just inferior to the subglottis, abutting the anterior tracheal wall (Figure 3). Optical alligator forceps were used to successfully extract the foreign body along with adjacent mild granulation tissue en bloc (Figure 4). Repeat bronchoscopy demonstrated mild subglottic irritation, friable tissue with a shallow linear ulcer along the right subglottis, and vocal fold edema. The tracheal rings distal to the foreign body site were well defined, with a sharp carina and clear bilateral mainstem bronchi. No additional foreign bodies were identified.

The patient tolerated the procedure without complications and was extubated without difficulty. Postoperatively, he was monitored and given intravenous dexamethasone for airway edema. He remained stable on room air, tolerated a regular diet, and had no stridor, wheezing, dyspnea, or cough. His voice returned to baseline per family report.

**Figure 5:**
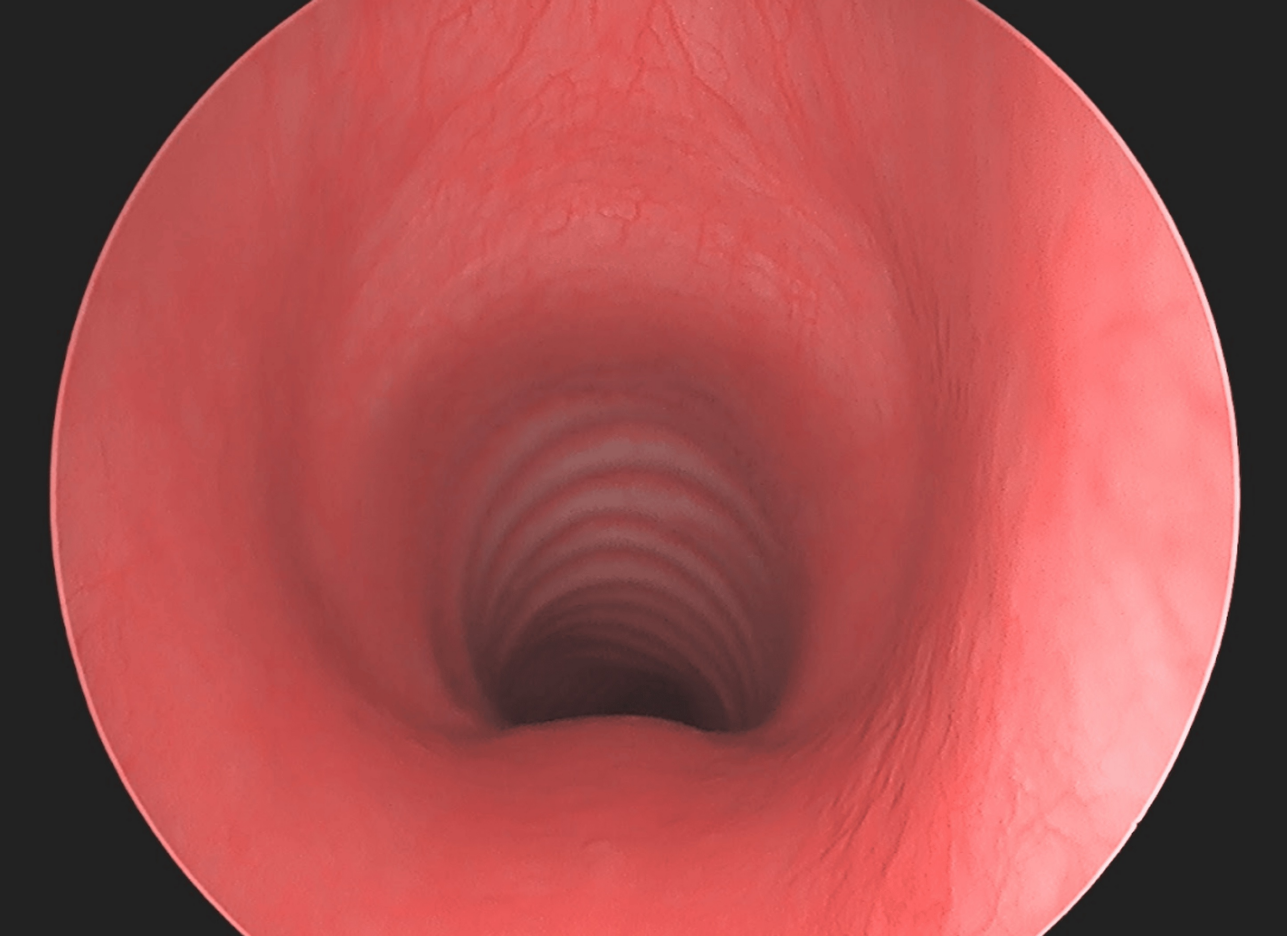
Bronchoscopic image at three-month follow-up revealing healthy tissue. The subglottis is completely healed with no evidence of scar tissue.

The patient was deemed appropriate for discharge home on postoperative day one with instructions to return for any recurrent respiratory symptoms, difficulty swallowing, or voice changes. Follow-up at two weeks revealed no airway symptoms and good exercise tolerance. Repeat bronchoscopy three months after the initial presentation revealed a normal, well-healed subglottis (Figure 5).

## Discussion

This case highlights several diagnostic pitfalls in evaluating suspected AFB, particularly in adolescents presenting subacutely with initially nondiagnostic imaging and outpatient discharge. Although more typically seen in younger children with small food items such as nuts and seeds, AFB in older children may present with more subtle symptoms due to greater physiological compensation, which may obscure the clinical picture [1]. In the presented patient, the absence of overt respiratory distress, normal oxygenation, and tolerance of oral intake contributed to an initially reassuring assessment, despite persistent symptoms.

Radiographic limitations contributed to the delayed identification of AFB. Radiolucent inorganic objects, such as plastic, evade detection on standard radiographs, and initial CT interpretation may be influenced by clinical framing, despite greater diagnostic sensitivity. In this case, the airway was initially not the primary focus, with imaging directed toward evaluation of esophageal impaction.

AFB and esophageal foreign body (EFB) share a similar initial presentation and follow a three-phase clinical course. The impaction phase is characterized by choking, gagging, a paroxysm of coughing, and acute distress at the time of the event, followed by an asymptomatic phase in which the foreign body lodges, reflexes fatigue, and symptoms subside. Finally, patients enter the complications phase, in which symptoms recur due to obstruction, erosion, or infection [4]. Patients may present during any of the three phases, making it difficult to distinguish between AFB and EFB. In adolescents capable of providing a history of the event, embarrassment or symptom minimization may further obscure the true clinical picture.

The location of the AFB in this case further contributed to an atypical presentation. Upper AFBs account for approximately 14.5% of all AFB cases compared to 83.6% of AFBs found in bronchial locations [5]. Of laryngotracheal foreign bodies, only 6.1% are estimated to involve the subglottic region [6]. In the presented case, the rigid contour of the bottle cap, positioned against the anterior tracheal wall, permitted partial airflow, thereby explaining the patient’s stable respiratory status. However, endoscopic findings of granulation tissue with mucosal ulceration indicate that localized inflammatory changes can develop within days, increasing the risk of progressive edema or acute deterioration. Patients with subglottic impaction may experience evolving pathology depending on the degree of obstruction and local inflammatory response, which may explain the progressive symptoms seen in our case.

In this patient, the retained bottle cap was ultimately visualized on bone windows of the neck CT upon re-evaluation prompted by additional negative esophageal and chest imaging. Given the difficulty of diagnosing AFB based on symptoms and radiographic findings alone, persistent symptoms following a suspected foreign body event warrant urgent endoscopic evaluation, including consideration of airway endoscopy even when initial esophageal evaluation or imaging is unrevealing. This approach is particularly important in patients with ongoing globus sensation, dysphagia, cough, or progressive respiratory symptoms despite reassuring initial findings. Delayed bronchoscopy beyond 24 h after aspiration has been associated with complication rates approaching 100%, compared to 22% when bronchoscopy is performed within 24 h [7].

Operative management in this case demonstrates other technical considerations. Direct laryngoscopy and bronchoscopy provided optimal exposure of the supraglottic and subglottic airway, allowing controlled visualization and safe extraction of AFB. Given the relatively narrow caliber of the subglottic airway, preparation for potential airway compromise, including severe hypoxemia, laryngospasm, and reactive edema following manipulation, is warranted, with preoperative discussion of possible emergency tracheotomy [8]. Perioperative corticosteroids may reduce the risk of postoperative airway edema following subglottic instrumentation, and are commonly described in perioperative management strategies for pediatric AFB removal [9,10].

## Conclusions

A normal physical examination and negative initial imaging do not reliably exclude AFB, particularly in patients with a clear history of aspiration accompanied by persistent or evolving symptoms. Radiolucent foreign bodies may evade detection on standard imaging and contribute to delayed diagnosis. In patients with ongoing symptoms after suspected foreign body ingestion or aspiration, standard management should include prompt endoscopic evaluation with consideration of airway-directed endoscopy, even in clinically stable pediatric patients.
